# Meteorological Register

**Published:** 1814-07

**Authors:** C. Blunt

**Affiliations:** Philosophical Instrument Maker, No. 38, Tavistock-Street, Covent-Garden


### METEOROLOGICAL REGISTER.

*From May the 25th, to June the 25th,* 1814.

Kept by C. BLUNT, Philosophical Instrument Maker, No. 38, Tavistock-Street, Covent-Garden.

| Moon. | Day. | Wind. | Barometrical Pressure. Max. | Min. | Mean. | Temperature. Max | Min. | Mean. | |
|---|---|---|---|---|---|---|---|---|---|
| ◐ | 26 | N | 29·96 | 29 94 | 29·95 | 58 | 38 | 45·66 | Fair |
| | 27 | NW | 29·90 | 29·77 | 29·83 | 65 | 44 | 53·3 | Fair |
| | 28 | NW | 29 71 | 29 65 | 29·675 | 69 | 44 | 53·3 | Fog at Night Fair |
| | 29 | N | 29·94 | 29·80 | 29·862 | 70 | 45 | 56·6 | Fair |
| | 30 | N | 30 07 | 30·07 | 30·07 | 56 | 48 | 52 | Fair |
| | 31 | E | 30 03 | 30·— | 30·017 | 68 | 44 | 57 | Fair |
| | 1 | SE | 30·03 | 29·90 | 29·955 | 67 | 47 | 55 | Rain |
| | 2 | SW | 29 87 | 29 84 | 29·862 | 68 | 49 | 57·3 | Rain |
| ○ | 3 | SE | 29·81 | 29·72 | 29·747 | 67 | 44 | 55 6 | Rain |
| | 4 | SE | 29·86 | 29·76 | 29·792 | 62 | 43 | 51 6 | Rain |
| | 5 | E | 30.17 | 30·01 | 30 135 | 60 | 45 | 51 3 | Cloudy |
| | 6 | NE | 30·10 | 30·07 | 30·112 | 60 | 42 | 51 | Cloudy |
| | 7 | SE | 30·02 | 30·— | 30·— | 58 | 40 | 49·6 | Cloudy |
| | 8 | E | 30·— | 29·95 | 29 957 | 60 | 44 | 52·3 | Fair |
| | 9 | EbyS | 30·06 | 30·01 | 30·042 | 59 | 40 | 51 6 | Fair |
| ◑ | 10 | E | 30 08 | 30·02 | 30 065 | 61 | 42 | 64 6 | Fair |
| | 11 | SE | 30 03 | 29·93 | 29 975 | 64 | 45 | 56·3 | Fair |
| | 12 | W | 30 — | 29·80 | 29·9 | 72 | 49 | 60 | Fair |
| | 13 | SE | 30·10 | 29·97 | 30·032 | 69 | 58 | 6[illegible] 6 | Rain |
| | 14 | SW | 30·07 | 30·02 | 30·057 | 76 | 60 | 67 6 | Fair |
| | 15 | SW | 29·92 | 29·86 | 29 89 | 80 | 53 | 67 60 | Rain |
| | 16 | SW | 30 — | 29·93 | 29 962 | 76 | 40 | 59 6 | Smal Rain a.m Fair p.m. |
| ● | 17 | NW | 30·10 | 30·04 | 30·072 | 70 | 42 | 57·3 | Fair |
| | 18 | N | 30·09 | 30·09 | 30·09 | 69 | 46 | 53 | Rain |
| | 19 | NW | 29·85 | 29·82 | 29 835 | 68 | 48 | 58 6 | Fair Showery |
| | 20 | N | 29·82 | 29·70 | 29·75 | 67 | 47 | 5 | Fair Cloudy |
| | 21 | N | 29·83 | 29·70 | 29·785 | 59 | 48 | 57 3 | Fair Cloudy |
| | 22 | E | 29·99 | 29 89 | 29 947 | 58 | 48 | 57 | Cloudy |
| | 23 | NW | 30 16 | 30·05 | 30·105 | 57 | 47 | 52·3 | Cloudy |
| ◐ | 24 | NW | 30·28 | 30·17 | 30·313 | 57 | 45 | 51 6 | Cloudy |
| | 25 | NW | 30·26 | 30·25 | 30·25 | 57 | 50 | 54 | Cloudy |

RESULTS.

Mean barometrical pressure 29·688
Maximum 30·28 wind at NW
Minimum 29·65 ——— NW

Mean temperature 55 96 deg.
Maximum 80, the wind at SW
Minimum 38, ——— N

*Scale exhibiting the prevailing Winds during the Month.*

| N | NE | E | SE | S | SW | W | NW |
|---|---|---|---|---|---|---|---|
| 5 | 1 | 6 | 6 | 0 | 4 | 2 | 7 |

| | Mean barometrical pressure. | Mean temperature. |
|---|---|---|
| From the first quarter on the 26th May, to the full moon on the 3d June | 29·902 | 54·02 |
| ——— full moon, to the last quarter on the 10th | 29·969 | 51 71 |
| ——— last quarter, to the new moon on the 17th | 29 983 | 62·47 |
| ——— new moon, to the first quarter on the 24th | 29·94 | 56·35 |

*Observations.*

*Observations.*—The only observation of importance to make, is on the general low temperature of the month, and the blight which prevails.

On the 14th, the first thunder-storm of the season occurred, with considerable severity. Lightning was vivid and frequent during the whole evening in the SW., and a heavy storm came on from the same quarter at about three, a. m. of the following morning; heavy rain accompanied it, and the thermometer rose during the storm from 59 to 67 degrees, falling to the former temperature at its close.

Measles and hooping-cough are very prevalent. Rheumatism and inflammatory sore-throat have not been unfrequent; and the class of pulmonary affections, notwithstanding the advanced period of the season, is still considerable.

